# The Four Years’ Curriculum

**Published:** 1915-06

**Authors:** E. C. Kirk


### THE FOUR YEARS' CURRICULUM.

BY E. C. KIRK, D.D.S., S.C.D.

After years of agitation and the educative effect of experience, which have fully demonstrated the inadequacy of the standard three years' curriculum as preparation for dental practice, the sentiment of the dental profession in America seems to be ready to support an increase in the time of the dental curriculum to four years. Indeed, the National Dental Association, the National Association of Dental Examiners, the National Association of Dental Faculties, and the Dental Faculties Association of American Universities, have each and all officially committed themselves by resolution as favorable to the advanced standard.

Dental educators have for years felt that the three years' curriculum was overcrowded to a degree which made efficient teaching in all of the required subjects of the curriculum almost impossible. Boards of examiners who have had to pass upon the products of our educational system under the three years' standard have complained of the insufficient preparation of candidates—all of which, with the steadily increasing data which make up the sum total of dental knowledge required for successful practice, has combined to make the demand for additional time in preparation for dental practice imperative.

In anticipation of the official increase in time of the dental course, much attention has been given to the consideration of the manner in which the added time should be utilized, and a variety of suggestions to that end have been made, among the most striking of which are suggestions which have been variously proposed for the incorporation in the new curriculum of cultural studies such as languages, rhetoric, history, economics, psychology, ethics, the principles of art, public speaking, freehand drawing, and the like. These suggestions have been made not only in good faith, but in clear recognition of an existing cultural need in the average dental student. No one can successfully question for a moment the immense value and utility of these cultural subjects as part of the education of the dentist, and it is doubtless because so many dental students are defective in the culture which these studies are believed to develop, and do develop when they are faithfully and intelligently pursued, that dental educators, realizing the intellectual deficiency which lack of training in these subjects entails, have proposed to remedy the defect by incorporating them into the dental curriculum.

We are in sympathetic agreement with those who realize the need of proper cultural development of the dental student, but we are wholly opposed to the means suggested for obtaining that kind of cultural training. The only logical way to obtain it is to demand proficiency in the group of subjects referred to as a part of the entrance qualification required for admission to the dental course.

In one of the proposed curricula published in this issue, forty-eight hours are given to materia medica and therapeutics, while to anatomy and its subdivisions are assigned a total of four hundred and forty-eight hours in this arrangement of time. Not only is undue emphasis given to anatomical and histologic study out of all relative proportion to its importance in the dental curriculum, but on the other hand, materia medica and therapeutics, which should involve adequate training in pharmacology, is minimized to a degree which would render the education of the dental student in that subject well-nigh worthless. Similarly, ninety-six hours are assigned to general bacteriology, with no provision for the special bacteriology of the mouth and its relations to mouth infections and systemic diseases. It is doubtless valuable and important for a dentist to know something if not all about the bacterial examination of water and the purification of sewage, but it is more important for him to know a great deal about the microbiology of the mouth and the bearings of that subject upon the question of local systemic disease. It is questionable whether in sixteen hours a student can secure more than a superficial smattering of dental metallurgy, and it is equally questionable whether in one hundred and fifty hours of combined lectures, laboratory, and practice he can secure more than a superficial smattering of orthodontia.

These instances are referred to for the purpose of pointing out the necessity not only for a complete readjustment of the details of the standard dental curriculum, but to emphasize the fact that in the readjustment it is of fundamental importance that the content, the extent, and the systematic grading of the course should be done with an eye single to the creation of intelligent and efficient practitioners of dentistry. With that object as the goal of dental education clearly in mind, the question of what constitutes the intelligent and successful practice of dentistry becomes the underlying factor of the whole educational scheme. It must be manifest to everyone who has kept in touch with the progress of dental and medical thought that the problems which today call for solution as presented by the *clientèle* of the average dentist are wholly different to those which confronted the profession less than a quarter of a century ago, and that they imperatively demand, in order to meet them, a correspondingly adapted type of dental practitioner. The immense possibilities which have been opened up, the recognition of the importance of mouth hygiene, the relation of oral infections to the bodily health, the nervous relations of defects in the dental mechanism, all present problems for solution which did not confront the earlier practitioners of dentistry, because the existence of such relations was then undreamed-of; in consequence of which dentistry developed almost wholly as a mechanical art, and the system of education related to dentistry was, until recently, almost wholly technical and manipulative in its ideals.

While no less emphasis should be placed upon that aspect of our educational work, it is undeniable that the point of dental attack upon the problem of disease has completely changed in the light of the developments of modern bacterio-pathology, neurology, and internal medicine; and in order that the future practitioner may hope to cope successfully with the health conditions involved within his sphere of practice, it is necessary that the time which is soon to be added to the standard dental curriculum shall be utilized for the more thorough training of students in the medical sciences that have direct application to his needs as a practitioner. This does not necessarily mean that we must import into the dental curriculum more of medicine, but it does mean that we shall more efficiently and broadly teach those phases of medical science which are directly applicable to our needs.

For example, the subjects of anatomy, physiology, and pathology, as ordinarily taught in the standard medical course, need to be so modified in their presentation with respect to the needs of the dental practitioner that he may be able to learn intensively those things which shall be of most use to him in his practice, and *extensively* those things that are of relatively minor importance. Our literature teems with enthusiastic statements of the ''wonderful progress of dentistry''; it teems almost as much with expressions of the desire for ''recognition,'' particularly recognition by the medical profession. With the opportunity for enlargement of the standard dental curriculum now confronting us, there is danger that we may fail to ''progress'' toward the goal of highest efficiency in the service of the community as dental practitioners, and because of our desire for ''recognition'' by the medical profession may overdo the arrangement of the new curriculum upon the medical side, by importing into it not simply that quantity and kind of medical science most useful and necessary for our needs, but that which has been found essential or desirable in the training of the medical practitioner. Our safety lies in a recognition of the soundness of the inquiry formulated years ago by Herbert Spencer, ''What knowledge is of most worth?''—and the knowledge of most worth to the practitioner of dentistry is the kind which develops his highest efficiency in the service of the public within the range of activities that he is called upon to fulfil.—*Cosmos* for May.

